# National Use of Safety-Net Clinics for Primary Care among Adults with Non-Medicaid Insurance in the United States

**DOI:** 10.1371/journal.pone.0151610

**Published:** 2016-03-30

**Authors:** Oanh Kieu Nguyen, Anil N. Makam, Ethan A. Halm

**Affiliations:** 1 Department of Internal Medicine, UT Southwestern Medical Center, Dallas, Texas, United States of America; 2 Department of Clinical Sciences, UT Southwestern Medical Center, Dallas, Texas, United States of America; Geisel School of Medicine at Dartmouth College, UNITED STATES

## Abstract

**Objective:**

To describe the prevalence, characteristics, and predictors of safety-net use for primary care among non-Medicaid insured adults (i.e., those with private insurance or Medicare).

**Methods:**

Cross-sectional analysis using the 2006–2010 National Ambulatory Medical Care Surveys, annual probability samples of outpatient visits in the U.S. We estimated national prevalence of safety-net visits using weighted percentages to account for the complex survey design. We conducted bivariate and multivariate logistic regression analyses to examine characteristics associated with safety-net clinic use.

**Results:**

More than one-third (35.0%) of all primary care safety-net clinic visits were among adults with non-Medicaid primary insurance, representing 6,642,000 annual visits nationally. The strongest predictors of safety-net use among non-Medicaid insured adults were: being from a high-poverty neighborhood (AOR 9.53, 95% CI 4.65–19.53), being dually eligible for Medicare and Medicaid (AOR 2.13, 95% CI 1.38–3.30), and being black (AOR 1.97, 95% CI 1.06–3.66) or Hispanic (AOR 2.28, 95% CI 1.32–3.93). Compared to non-safety-net users, non-Medicaid insured adults who used safety-net clinics had a higher prevalence of diabetes (23.5% vs. 15.0%, p<0.001), hypertension (49.4% vs. 36.0%, p<0.001), multimorbidity (≥2 chronic conditions; 53.5% vs. 40.9%, p<0.001) and polypharmacy (≥4 medications; 48.8% vs. 34.0%, p<0.001). Nearly one-third (28.9%) of Medicare beneficiaries in the safety-net were dual eligibles, compared to only 6.8% of Medicare beneficiaries in non-safety-net clinics (p<0.001).

**Conclusions:**

Safety net clinics are important primary care delivery sites for non-Medicaid insured minority and low-income populations with a high burden of chronic illness. The critical role of safety-net clinics in care delivery is likely to persist despite expanded insurance coverage under the Affordable Care Act.

## Introduction

The U.S. health care ‘safety-net’ is a fragmented and heterogeneous network of public hospitals, clinics, community health centers (CHCs), and other healthcare organizations defined only by their shared mission–to provide care to individuals regardless of ability to pay.[1] The safety-net has a well-recognized and critical role as a provider of ‘last resort’ for 44 million uninsured and underserved Americans, disproportionately minorities, immigrants, Medicaid beneficiaries, and those in disadvantaged communities–vulnerable populations who cannot afford to seek care elsewhere. In contrast, individuals with non-Medicaid primary health insurance coverage–i.e., those with private insurance or Medicare–are thought to be less reliant on safety-net providers given their potential access to care in other settings.[2] With evidence suggesting lower quality and lower patient satisfaction with care in safety-net settings, many anticipate that both newly and previously insured individuals will choose to seek care elsewhere.[2,3]

However, given the unique challenges of caring for vulnerable populations, safety-net providers are thought to have a comparative advantage in addressing unmet social needs related to language, culture, employment, and transportation, compared to non-safety-net providers.[1,3,4] Further, unmet social needs are not limited to low-income individuals and have been previously related to high healthcare utilization and costs.[5–8] Consequently, the safety-net may also have an important but underrecognized role as a regular source of care for non-Medicaid insured individuals with these specific needs. Among CHCs–one of several types of safety-net outpatient settings–national estimates suggest that up to one-quarter of individuals cared for each year are insured through private insurance or Medicare.[9] Prior studies suggest that non-Medicaid insured individuals may seek care in the safety-net due to lack of access, but may also do so for unrelated reasons including proximity, affordability and the ability to better meet specialized needs compared to other providers.[10–12] However, these studies were limited to non-Medicaid insured populations in single states. To date, there have been no comprehensive national studies assessing the role of the safety-net in caring for non-Medicaid insured populations, nor assessing the potentially distinct characteristics and care needs of these individuals.

To address this gap in the literature, we used nationally representative data from the National Ambulatory Medical Care Surveys (NAMCS), which have more systematically included information on the use of safety-net clinics since 2006. Our objective was to assess the national prevalence and characteristics of insured individuals using safety-net settings for primary care. We hypothesized that contrary to popular belief, a substantial number of visits to safety-net clinics are by patients with non-Medicaid insurance, and that higher-need insured individuals, with a greater burden of illness and clinical complexity, are more likely to receive regular care in the safety-net. Greater understanding of the role of the safety-net in caring for non-Medicaid insured individuals is critical to informing the national debate on the appropriate role for and financing of safety-nets in the era of insurance expansion and delivery system reform.

## Methods

### Data and Study Design

We analyzed NAMCS from 2006 to 2010. NAMCS is an annual, nationally representative cross-sectional survey of ambulatory office visits administered by the National Center for Health Statistics (NCHS). NAMCS uses a multi-stage probability sampling design to represent all visits to non-federally employed office-based physicians in the United States engaged in direct patient care.[13] Trained clinic staff collect data for all visits during a one-week assigned reporting period with oversight from the U.S. Bureau of the Census. A more detailed description of the NAMCS methodology is available from the NCHS.[14–17]

### Study Population

We included all primary care visits by individuals 18 years or older with private insurance or Medicare as the expected primary payer, since individuals with these forms of insurance have the greatest choice in where they receive their care.[18,19] We excluded individuals with Medicaid as a primary payer given significant state-level variability in benefits, which may result in more limited access to primary care compared to private insurance or Medicare.[20] Because all potential payers are recorded for each visit in NAMCS, we defined the expected primary payer using the following hierarchy of payment categories: Medicare, private insurance, Medicaid, other. Of note, NAMCS does not distinguish Medicare or Medicaid managed care from traditional Medicare and Medicaid. Primary care visits were defined by NAMCS as visits with family medicine, general internal medicine, preventive medicine, obstetrics and gynecology, hospice and palliative care, general practice, and geriatrics providers.[21]

### Primary Outcome

The primary outcome was whether a primary care visit occurred in a safety-net clinic. We broadly defined ‘safety-net clinic’ as a community health center (CHC) or a non-federal government-run clinic given that these both are ‘core safety-net providers’ as defined by the Institute of Medicine. Because safety-net clinics are defined by mission rather than a regulatory definition, many are not necessarily designated CHCs, though the majority are operated by non-federal public agencies, such as city and county governments.[1] CHCs were identified by NCHS for sampling in NAMCS starting in 2006 and included federally qualified health centers (FQHC), Urban Indian Health Programs with FQHC designation, and FQHC look-alikes (organizations that meet the eligibility requirements for FQHCs and receive cost-based reimbursement but do not otherwise receive funding under Section 330 of the Public Health Services Act).[21,22]

### Demographic, Visit and Clinical Characteristics

In addition to primary payer, we defined secondary payers as any additional listed potential payers according to the same hierarchy. We defined ‘dual eligibles’ as individuals with Medicare and Medicaid as primary and secondary payers respectively.

Race/ethnicity were defined based on imputed fields in NAMCS, since these data were missing for 22–35% of records per year from 2006–2010 (an approach used in previously published studies).[21] Zip code-level socioeconomic variables were determined by the NCHS using 2000 U.S. Census data.[23] Education was defined by the U.S. Census Bureau as prevalence of those with a bachelor’s degree within a given ZIP code; cut-points were based on population quartiles for the area measure. Poverty was defined by the U.S. Census Bureau as the prevalence of persons below the federal poverty level within a given ZIP code; cut-points for categorization of poverty prevalence were defined as per the Public Health Geocoding Disparities Project. Areas with ≥20% poverty were considered ‘high poverty.’[24,25] Metropolitan statistical areas (MSAs) were defined per the U.S. Office of Management and Budget. We defined ‘rural areas’ as non-MSA areas, consistent with the definition used by the Federal Office of Rural Health Policy.[26]

The presence of up to 14 different chronic medical conditions was recorded, independent of visit diagnoses.[21] Up to eight medications maximum were recorded per visit.[21] We defined ‘multimorbidity’ as the presence of two or more chronic conditions and ‘polypharmacy’ as four or more medications, consistent with prior literature.[27,28]

### Statistical Analysis

All analyses accounted for the complex survey design and used visit, strata, and primary sampling unit design weights provided by NAMCS to reflect national estimates. We conducted all analyses using Stata 12.0 (StataCorp, College Station, Texas). We estimated prevalence of primary care visits in the safety-net using weighted percentages and estimated absolute number of visits per year. We used linear regression with visit year as an ordinal predictor to test for linear trends in safety-net use during the study period. We conducted bivariate and multivariate logistic regression analyses to examine characteristics associated with safety-net use. Due to differences in age distribution among individuals with private insurance versus Medicare, we examined bivariate associations between age and safety-net use separately in these subgroups. Because accounting for the interaction between age and payer did not meaningfully change our findings, we opted to omit this interaction term in our multivariate model for ease of interpretation. The final multivariate model included all sociodemographic characteristics including age, sex, race/ethnicity, dual eligible status, rural area status, geographic region, prevalence of poverty in zip code, prevalence of bachelor’s degree in zip code; clinical characteristics including polypharmacy, multimorbidity, frequent visits (≥5 in the past 12 months); and chronic conditions with p-values ≤0.05 from bivariate analyses. Due to potential co-linearity between polypharmacy, frequency of visits, multimorbidity, and significant chronic conditions, we performed sensitivity analyses assessing each variable independently in the multivariate model and found no meaningful difference in our findings (data not shown).

Model fit was assessed using an extension of the Hosmer-Lemeshow goodness-of-fit test for survey weighted data (p = 0.35, suggesting no evidence of lack of fit).[29] Given the large proportion of imputed race/ethnicity data, we performed a sensitivity analysis using only non-imputed values. Our findings were robust to the exclusion of imputed data (data not shown). We performed a subgroup analysis to identify predictors of safety-net use specifically among dual eligibles, given that this population has a higher burden of illness, disability and social disadvantage.[30]

This study was deemed exempt from review by the UT Southwestern Medical Center institutional review board.

## Results

**Fig 1:**
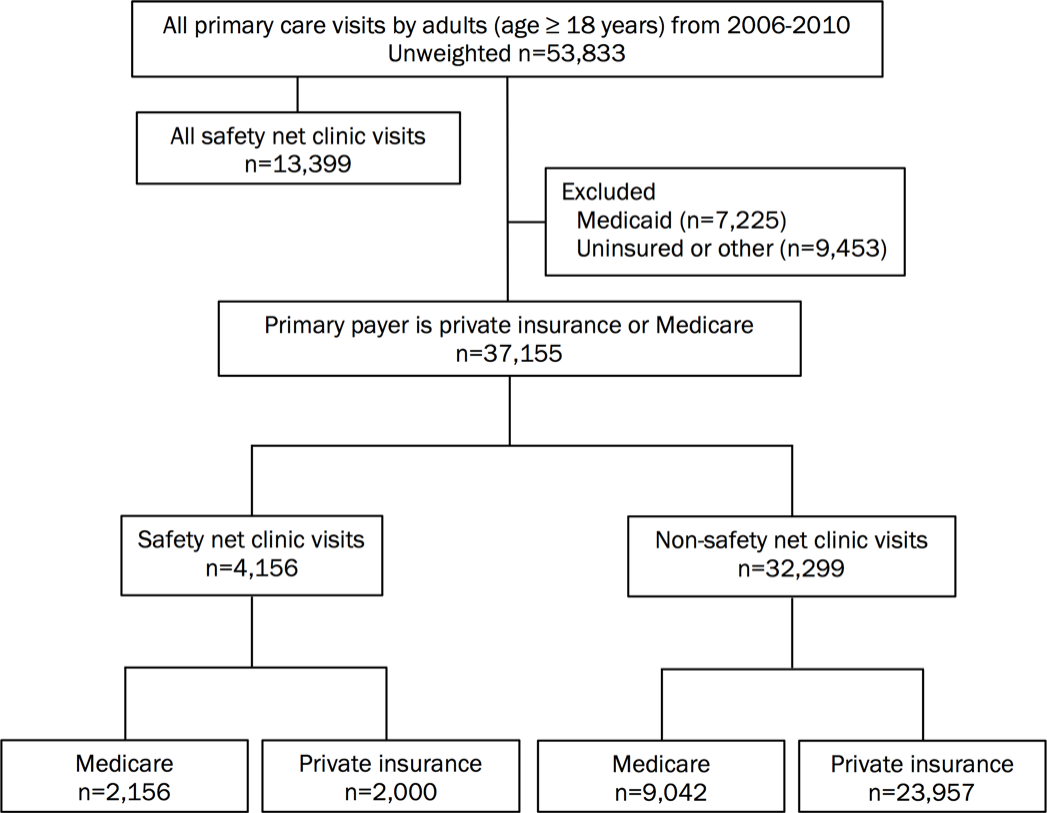
Study Flowchart.

From 2006 to 2010, NAMCS included a total of 53,833 visits by adults to primary care clinics. Of these, 37,155 visits were by individuals with either Medicare or private (i.e., non-Medicaid) insurance (Fig 1). Among individuals with non-Medicaid insurance, 4,156 visits were to a safety-net clinic (2.0% of weighted visits).

The 4,156 safety-net clinic visits among non-Medicaid insured adults represent an estimated 35.0% of all safety-net clinic visits for primary care (n = 13,399), or 6,642,000 visits annually. Medicare beneficiaries and privately insured individuals accounted for 19.8% and 15.2% of safety-net visits respectively. We did not observe any temporal trend in safety-net clinic use among non-Medicaid insured individuals from 2006 to 2010 (OR 1.05, 95% CI 0.92–1.20, p = 0.44).

### Characteristics of Non-Medicaid Insured Safety-Net Users

Non-Medicaid insured individuals using the safety-net for primary care were more likely to be black (17.4% vs. 10.2%, p<0.001) or Hispanic (22.6% vs. 9.1%, p<0.001); dual eligible (16.3% vs. 2.0%) and be from areas with high rates of poverty and limited education compared to their non-safety-net counterparts (Table 1). Safety net users were also more likely to be from the Northeast or West.

**Table 1:** Demographic, Visit, and Clinical Characteristics among Non-Medicaid Insured Adults With a Primary Care Visit, Stratified by Safety-Net Clinic Use.

	Weighted % (SE)		
		Safety-Net (N = 4,156)	Non-Safety-Net (N = 32,999)	p-value
Estimated total population	6,642,000	324,961,000	
Demographic Characteristics			
Age, years, mean ± SD*			
Private insurance	42.7 ± 29.2	45.3 ± 12.1	0.002
Medicare	65.2 ± 28.3	72.3 ± 9.4	<0.001
Female	63.8 (1.7)	65.5 (0.6)	0.99
Race/ethnicity			<0.001
White, non-Hispanic	54.2 (4.2)	75.5 (1.2)	
Black, non-Hispanic	17.4 (3.2)	10.2 (0.9)	
Hispanic	22.6 (3.7)	9.1 (0.7)	
Other	5.8 (1.6)	5.3 (0.6)	
Primary payer			<0.001
Private insurance	43.4 (4.1)	70.5 (1.1)	
Medicare	56.6 (4.1)	29.5 (1.1)	
Dual eligible	16.3 (4.3)	2.0 (0.2)	<0.001
Rural area^†^	19.7 (7.1)	14.3 (3.8)	0.32
Geographic region			0.04
Northeast	26.4 (5.0)	16.2 (1.7)	
Midwest	13.0 (5.1)	24.3 (2.0)	
South	32.9 (6.8)	38.9 (2.5)	
West	25.7 (6.3)	20.6 (2.2)	
Prevalence of poverty in zip code^‡^			<0.001
Lowest (<5%)	3.6 (0.8)	23.6 (1.5)	
Low (5–9.9%)	15.7 (3.3)	30.9 (1.4)	
Moderate (10.0–19.9%)	37.3 (3.1)	29.0 (1.6)	
High (≥ 20%)	38.3 (4.0)	10.6 (0.8)	
Prevalence of bachelor’s degree in zip code			<0.001
Lowest (<12.8%)	46.3 (4.3)	20.4 (1.3)	
Low (12.8–19.7%)	23.3 (2.6)	22.3 (1.3)	
Moderate (19.8–31.7%)	16.8 (2.8)	24.7 (1.1)	
High (>31.7%)	8.5 (1.7)	26.8 (1.5)	
Visit Characteristics			
Seen in practice before	92.3 (1.3)	92.3 (0.4)	0.99
Frequent visits (≥5) in last 12 months	44.2 (4.0)	26.9 (0.7)	<0.001
Seen for care of a chronic problem	45.2 (2.7)	34.7 (0.9)	0.004
Clinical Characteristics			
Polypharmacy (≥4 total medications)	48.8 (4.9)	34.0 (0.9)	<0.001
Multimorbidity (≥2 chronic conditions)	53.5 (3.4)	40.9 (1.0)	<0.001
Types of chronic conditions			
Arthritis	16.3 (1.7)	15.0 (0.5)	0.43
Asthma	6.6 (1.0)	5.8 (0.2)	0.41
Cancer	3.4 (0.5)	4.2 (0.2)	0.18
Cerebrovascular disease	3.0 (0.6)	2.0 (0.1)	0.09
Chronic renal failure	1.7 (0.3)	1.5 (0.2)	0.65
Congestive heart failure	3.4 (0.7)	2.3 (0.1)	0.05^§^
Chronic obstructive pulmonary disease	7.8 (1.0)	5.8 (0.2)	0.02
Depression	14.0 (1.8)	11.0 (0.4)	0.07
Diabetes	23.5 (2.0)	15.0 (0.4)	<0.001
Hyperlipidemia	27.4 (2.7)	25.8 (0.9)	0.54
Hypertension	49.4 (3.4)	36.0 (0.8)	<0.001
Ischemic heart disease	5.7 (0.9)	4.2 (0.2)	0.06
Obesity	12.4 (1.6)	10.0 (0.4)	0.10
Osteoporosis	6.2 (1.1)	4.4 (0.2)	0.06

* Due to marked differences in age distribution among individuals with Medicare versus those with private insurance, we presented mean age ± SD separately for each subgroup.

^†^ Defined as areas categorized as non-metropolitan statistical areas (non-MSAs).

^‡^ Categories correspond to quartiles.

^§^ Rounded from p = 0.054; heart failure was not included in the multivariate model given p>0.05.

With regard to visit characteristics, 92.3% of all non-Medicaid insured safety-net users were seen before in the practice, confirming that these clinics were regular sources of care. Non-Medicaid insured safety-net users were more likely to have had five or more visits in the past twelve months (44.2% vs. 26.9%, p<0.001) and be seen for a chronic problem (45.2% vs. 34.7%, p = 0.004) compared to non-safety-net users. With respect to clinical characteristics, insured safety-net users had higher prevalence of polypharmacy (48.8% vs. 34.0%, p<0.001), multimorbidity (53.5% vs. 40.9%, p<0.001), diabetes (23.5% vs. 15.0%, p<0.001), hypertension (49.4% vs. 36.0%, p<0.001) and chronic obstructive pulmonary disease (7.8 vs 5.8%, p = 0.02).

### Predictors of Safety-Net Primary Care Clinic Use

Our adjusted analysis confirmed that minority race/ethnicity and residing in a high-poverty neighborhood were among the strongest predictors of safety-net use among insured individuals (Table 2). Age less than 65 years and being dually eligible for Medicare and Medicaid were also strong demographic predictors.

**Table 2:** Predictors of Safety-Net Use among Non-Medicaid Insured Adults.

	Odds Ratio (95% CI)	
	Unadjusted	Adjusted*
Age, years^†^		
18–64	—	2.44 (1.80–3.31)^‡^^,^ ^§^
≥ 65	—	[Reference]
Female	0.93 (0.80–1.07)	0.89 (0.73–1.07)
Race/ethnicity		
White, non-Hispanic	[Reference]	[Reference]
Black, non-Hispanic	2.39 (1.53–3.73)^§^	1.97 (1.06–3.66)^‖^
Hispanic	3.46 (2.27–5.29)^§^	2.28 (1.32–3.93)^‖^
Other	1.54 (0.88–2.69)	1.46 (0.56–3.80)
Dual eligible	5.54 (3.18–9.68)^§^	2.13 (1.38–3.30)^§^
Rural area	1.46 (0.68–3.11)	1.09 (0.47–2.52)
Geographic region		
Midwest	[Reference]	[Reference]
Northeast	3.03 (1.27–7.28)^‖^	5.87 (2.48–13.93)^§^
South	1.58 (0.63–3.95)	1.69 (0.70–4.10)
West	2.50 (1.13–5.54)^‖^	4.23 (1.83–9.76)^§^
Prevalence of poverty in zip code		
Lowest (<5%)	[Reference]	[Reference]
Low (5–9.9%)	3.39 (2.23–5.15)^§^	2.50 (1.34–4.68)^‖^
Moderate (10.0–19.9%)	8.53 (5.19–14.02)^§^	5.34 (2.78–10.25)^§^
High (≥ 20%)	23.93 (13.48–42.48)^§^	9.53 (4.65–19.53)^§^
Prevalence of bachelor’s degree in zip code		
Lowest (<12.8%)	7.21 (4.18–12.44)^§^	1.90 (0.96–3.78)
Low (12.8–19.7%)	3.31 (1.97–5.55)^§^	1.12 (0.60–2.09)
Moderate (19.8–31.7%)	2.16 (1.56–2.98)^§^	0.79 (0.50–1.27)
High (>31.7%)	[Reference]	[Reference]
Frequent visits (≥5) in past 12 months	2.15 (1.54–3.01)^§^	1.79 (1.14–2.81)^‖^
Polypharmacy (≥4 medications)	1.85 (1.28–2.68) ^‖^	1.64 (1.08–2.49)^‖^
Multimorbidity (≥2 chronic conditions)	1.69 (1.30–2.19)^§^	0.83 (0.59–1.16)
Chronic conditions		
Diabetes	1.74 (1.41–2.15)^§^	0.97 (0.76–1.23)
Hypertension	1.73 (1.34–2.24)^§^	1.40 (1.05–1.85)^‖^
Chronic obstructive pulmonary disease	1.37 (1.05–1.78)^‖^	1.16 (0.85–1.58)

* Adjusted for all characteristics listed in Table 1 and accounting for the complex survey sampling design.

^†^ For the unadjusted analysis, we evaluated age stratified by primary payer (see Methods for further details). For individuals with Medicare, age 18–64 was associated with a 3.40 higher odds of safety-net use (95% CI 2.63–4.41) compared to age ≥ 65 years. For privately insured individuals, age 18–64 years was associated with a 2.12 higher odds of safety-net use (95% CI 1.53–2.93) compared to age ≥ 65 years.

^‡^ The association of age with safety-net use for all insured individuals. We omitted the interaction term for age and payer from the adjusted model, given it did not meaningfully change our findings (see Methods for further details).

^§^ p≤0.001.

^‖^ p≤0.05.

We found significant regional variation in safety-net use among insured individuals. Specifically, being from the Northeast was associated with nearly a six-fold greater odds of safety-net use (AOR 5.87, 95% CI 2.48–13.93) and being from the West as associated with four-fold greater odds of safety-net use (AOR 4.23, 95% CI 1.83–9.70) compared to being from the Midwest.

We also found that certain clinical characteristics were robust predictors of safety-net use. Having five or more visits in the prior year (AOR 1.79, 95% CI 1.14–2.81), and the presence of polypharmacy (AOR 1.64, 95% CI 1.08–2.49) or hypertension (AOR 1.40, 95% CI, 1.05–1.85) were independently associated with safety-net use. However, after adjustment, multimorbidity, diabetes, and chronic obstructive pulmonary disease were no longer significant predictors.

### ‘Dual Eligibles’

Nearly one-third (28.9%) of Medicare beneficiaries in the safety-net were dual eligibles, compared to only 6.8% of beneficiaries not in the safety-net (p<0.001). As a whole, dual eligibles were significantly older than non-dual eligibles (64.0±16.0 years vs. 53.1±15.6 years, p<0.001). Other characteristics among dual eligibles in the safety-net were otherwise similar to those observed for the overall population of insured safety-net users (S1 Table). Predictors of safety-net use among this population were also similar to those identified among the overall insured population, except that osteoporosis and not diabetes or hypertension was a clinical predictor of safety net use; race/ethnicity and measures of socioeconomic status had a markedly attenuated association; and age less than 65 years was not associated with increased safety-net use (Table 3).

**Table 3:** Predictors of Safety-Net Use among Dual Eligible Individuals.

	Odds Ratio (95% CI)	
	Unadjusted	Adjusted*
Age, years		
18–64	0.65 (0.42–1.02)	0.86 (0.59–1.25)
≥ 65	[Reference]	[Reference]
Female	0.95 (0.64–1.40)	0.86 (0.54–1.39)
Race/ethnicity		
White, non-Hispanic	[Reference]	[Reference]
Black, non-Hispanic	1.10 (0.46–2.63)	1.44 (0.63–3.28)
Hispanic	4.31 (2.05–9.07)^†^	1.90 (0.91–3.97)
Other	2.01 (0.54–7.43)	0.89 (0.23–3.39)
Prevalence of poverty in zip code		
Lowest (<5%)	[Reference]	[Reference]
Low (5–9.9%)	1.10 (0.35–3.45)	1.20 (0.29–4.90)
Moderate (10.0–19.9%)	1.30 (0.43–3.89)	1.29 (0.33–5.12)
High (≥ 20%)	4.97 (1.27–19.37) ^‡^	3.46 (0.77–15.47)
Prevalence of bachelor’s degree in zip code		
Lowest (<12.8%)	2.08 (0.68–6.36)	1.08 (0.41–2.84)
Low (12.8–19.7%)	0.86 (0.32–2.32)	0.57 (0.20–1.64)
Moderate (19.8–31.7%)	0.71 (0.35–1.44)	0.59 (0.25–1.4)
High (>31.7%)	[Reference]	[Reference]
Rural area	0.43 (0.15–1.21)	1.21 (0.49–2.98)
Geographic region		
Midwest	[Reference]	[Reference]
Northeast	11.69 (3.71–36.80)^†^	10.37 (3.51–30.59)^†^
South	2.19 (0.88–5.43)	1.67 (0.62–4.55)
West	12.34 (4.02–37.85)^†^	9.36 (3.05–28.74)
Frequent visits (≥5) in past 12 months	2.18 (0.83–5.75)	2.19 (1.09–4.40)^‡^
Polypharmacy (≥4 medications)	2.50 (0.97–6.45)	2.26 (1.04–4.92)^‡^
Multimorbidity (≥2 chronic conditions)	1.19 (0.72–1.95)	0.81 (0.44–1.51)
Chronic conditions		
Chronic obstructive pulmonary disease	0.52 (0.23–0.93)^‡^	1.02 (0.56–1.86)
Hypertension	1.69 (1.11–2.57)^‡^	1.33 (0.82–2.15)
Osteoporosis	2.14 (1.15–3.98)^‡^	2.32 (1.15–4.68)^‡^

* Adjusted for all characteristics listed in Table 1 and accounting for the complex survey sampling design.

^†^ p≤0.001.

^‡^ p≤0.05.

## Discussion

To our knowledge, this is the first comprehensive national study of safety-net use among non-Medicaid insured adults. We found that over one-third of primary care visits in safety-net settings occurred among adults with non-Medicaid insurance. This was surprising, given that safety-net clinic visits only account for 2% of all primary care visits in this population. Despite being younger than their non-safety-net counterparts, safety-net users tended to have higher rates of chronic disease and polypharmacy. The strongest predictors of safety-net use among adults with non-Medicaid insurance were being from high-poverty neighborhoods, being from the Northeast or West, being black or Hispanic, being a dual eligible, being younger than age 65, having frequent visits in the prior year, and having polypharmacy or hypertension. Nearly one-third of Medicare beneficiaries who were safety-net users were dual eligibles (i.e., had Medicaid as secondary insurance). Dual eligibles have the same potential access to primary care providers but have more complex care needs and higher health utilization than other Medicare beneficiaries.[31]

Our findings underscore the important but underrecognized role of safety-net clinics as primary care homes for insured individuals with private insurance or Medicare, who account for a disproportionate share of safety-net utilization. Though they account for 35% of safety-net visits overall (and 34% of CHC visits specifically) they comprise only 25% of total patients and 15% of total national annual revenue in CHCs, the only outpatient safety-net setting for which national data exists.[9,32,33] Although private insurers and Medicare are preferred third-party payers outside of the safety-net, both reimburse less than Medicaid for services rendered in CHCs.[12,34,35] These unfavorable reimbursement rates often do not cover the cost of even a single visit in a CHC, nor the cost of essential ancillary services (i.e., case management) for individuals with non-Medicaid insurance, that are often otherwise unavailable outside of safety-net settings.[12,34,35]

Supporting the hypothesis that non-Medicaid insured individuals in the safety-net have a greater burden of illness and clinical complexity compared to non-safety-net counterparts, we found that polypharmacy, hypertension, and frequent visits in the prior year were strong predictors of safety-net use. Though we were unable to directly assess the severity of individual chronic conditions using NAMCS data, we considered polypharmacy and frequency of visits as reasonable proxies given their associations with illness severity.[36,37] We suspect that multimorbidity was not a predictor of safety-net use given that the comorbidity count in NAMCS does not capture the presence of certain serious chronic conditions that disproportionately affect underserved populations such as human immunodeficiency virus, substance abuse, or mental illness.[38] The implications of our findings are that safety-net clinics disproportionately care for high-need non-Medicaid insured individuals. Notably, other studies have suggested that safety-net clinics may lack the resources [1,39–42] to invest in coordinated, multidisciplinary care models that are needed to achieve high quality, equitable, and comprehensive care.[12,34,43,44]

Our findings confirm that minority and low-income insured individuals with private insurance or Medicare are more likely to use safety-net providers, though it is unclear whether the reason for greater safety-net use is a matter of proximity, access, preference, affordability, loyalty, cultural concordance, or other considerations. Safety net providers are often located by design in ‘medically underserved’ areas; consequently, use of safety-net providers among insured minority and low-income individuals may in part reflect proximity and/or lack of easy access to alternate sites for primary care.[45,46] However, past studies have suggested that though proximity is an important predictor of safety-net use in rural areas, it is a much less important predictor in urban areas.[47] Moreover, our finding that being from the Northeast was a strong predictor of safety-net use–a region with lower rates of uninsurance, greater supply of private primary care providers, and lower number of CHCs than the West or the South—suggests that proximity and access are unlikely to be the sole determinants of safety-net use.[48,49]

Affordability may also be an important consideration for seeking care in the safety-net. Nearly 1 in 8 privately insured adults under 65 years old spend a high share of annual income on health expenses.[50] Financial strain due to health spending is likely even more pressing for Medicare beneficiaries, half of whom have an annual income under 200% of the federal poverty level.[51,52] We found that increasing prevalence of poverty was associated with increasing odds of safety-net use in a dose-dependent fashion. Additionally, dual eligible status–another strong predictor of safety-net use—may be an indirect indicator of individual income since Medicare beneficiaries are only eligible for Medicaid if they are extremely low-income or disabled. To qualify for Medicaid through disability, disabled individuals must also either receive cash assistance through Supplemental Security Income (SSI) or otherwise meet income eligibility criteria, which varies from state to state.[51] These findings support the notion that insured individuals with private insurance or Medicare may use the safety-net at least in part due to financial strain and increased affordability of services.

Our study has important implications for understanding the effect of expanded insurance coverage on the safety-net in a post-ACA environment. Expanded insurance coverage that nonetheless remains unaffordable due to high out-of-pocket costs may result in self-selected clustering of needier and sicker insured individuals in more affordable safety-net clinics. Many of the newly covered are likely to have previously gone without care while uninsured, and have higher health care use after receiving insurance coverage.[53,54] This would result in increasing strain on an already overburdened and underfunded safety-net, with worsening of already restricted access to specialty services, disruptions in care, and long waiting periods among safety-net patients, especially given the already anticipated increase in demand for safety-net services among individuals newly covered with Medicaid expansion.[55,56] Consequently, it is unlikely that expanding coverage will obviate the vital role of the safety-net in caring for minority, low-income, and high-need individuals, irrespective of insurance type. Rather, maintaining adequate support for a strong safety-net, in addition to expanding coverage, will be critical to ensuring optimal access and to addressing racial/ethnic and social class disparities in health care outcomes.[57]

Our results should be interpreted in light of several limitations. First, our definition of ‘safety-net clinics’ included only officially designated FQHCs/CHCs and non-federal government clinics and excluded many other de facto safety-net settings not captured in NAMCS including emergency and hospital outpatient departments.[1,45,58–60] Though some data on emergency and hospital outpatient departments are available through the National Hospital Ambulatory Medical Care Surveys, there are no designations to allow uniform identification of safety-net settings or visits for primary care. As such, we did not include this data in our analysis. Consequently, we anticipate that our underrepresentation of safety-net settings resulted in a conservative estimate of safety-net clinic use among insured individuals. Second, we did not have data on geographic proximity, physician language and race/ethnicity, availability of services, or linkages to community resources. These domains, as well as patient preferences, are likely to be important predictors of safety-net use among insured individuals and are key targets for future investigation.

## Conclusion

Non-Medicaid insured adults account for over one-third of primary care visits to safety-net clinics. Given the higher burden of illness and clinical complexity among non-Medicaid insured safety-net users, additional attention to national policy, financing, and support for the safety-net is needed to optimize its function as a medical home for many low-income and minority insured Americans.

## Supporting Information

S1 Table Characteristics of Dual Eligible Individuals with Primary Care Visits, Stratified by Safety-Net Clinic Use. (DOCX)
