# Supplementary material for: National Use of Safety-Net Clinics for Primary Care among Adults with Non-Medicaid Insurance in the United States
Source: PLoS One. 2016 Mar 30;11(3):e0151610. doi: 10.1371/journal.pone.0151610 (PMC4814117; doi:10.1371/journal.pone.0151610)
Supplement: S1 Table — (DOCX) [file pone.0151610.s001.docx]

| S1 Table. Characteristics of Dual Eligible Individuals with Primary Care Visits, Stratified by Safety-Net Clinic Use | | | |
| --- | --- | --- | --- |
|  | Weighted % (SE) | | p-value |
|  | Safety-Net Clinics  (N = 526) | Non-Safety-Net Clinics  (N = 706) |  |
| Estimated total population | 1,086,000 | 6,538,000 |  |
| Demographic characteristics |  |  |  |
| Age, years, mean ± SD | 66.1 ± 29.6 | 63.6 ± 15.9 | 0.23 |
| Female | 66.8 (3.8) | 68.0 (2.6) | 0.80 |
| Race/ethnicity |  |  | 0.01 |
| White, non-Hispanic | 35.8 (5.3) | 56.9 (3.8) |  |
| Black, non-Hispanic | 13.0 (5.3) | 18.7 (2.7) |  |
| Hispanic | 38.4 (9.9) | 14.2 (2.6) |  |
| Other | 12.9 (6.7) | 10.2 (3.4) |  |
| Rural area | 11.0 (5.9) | 22.5 (5.9) | 0.10 |
| Geographic region |  |  | <0.001 |
| Northeast | 27.1 (11.4) | 12.1 (2.4) |  |
| Midwest | 4.8 (2.2) | 25.1 (3.4) |  |
| South | 17.3 (6.8) | 41.3 (4.5) |  |
| West | 50.8 (15.3) | 21.6 (4.8) |  |
| Prevalence of poverty in zip code |  |  | <0.001 |
| Lowest (<5%) | 2.3 (1.3) | 5.2 (1.1) |  |
| Low (5-9.9%) | 10.8 (4.4) | 22.8 (2.9) |  |
| Moderate (10.0-19.9%) | 24.1 (5.9) | 43.2 (3.2) |  |
| High (≥ 20%) | 55.9 (10.5) | 26.1 (2.7) |  |
| Prevalence of bachelor’s degree in zip code |  |  | 0.10 |
| Lowest (<12.8%) | 57.7 (12.6) | 38.3 (3.7) |  |
| Low (12.8-19.7%) | 15.1 (6.3) | 24.2 (3.0) |  |
| Moderate (19.8-31.7%) | 10.6 (4.4) | 21.4 (2.5) |  |
| High (>31.7%) | 9.5 (3.9) | 13.3 (2.3) |  |
|  |  |  |  |
| Visit Characteristics |  |  |  |
| Seen in practice before | 97.4 (1.3) | 94.2 (1.1) | 0.18 |
| Frequent visits (≥5) in past 12 months | 62.5 (10.6) | 43.2 (2.7) | 0.11 |
| Seen for care of a chronic problem | 52.9 (5.2) | 47.5 (2.5) | 0.63 |
|  |  |  |  |
| Clinical Characteristics |  |  |  |
| Polypharmacy (≥4 total medications) | 74.3 (8.8) | 53.6 (3.3) | 0.05 |
| Multimorbidity (≥2 chronic conditions) | 74.9 (4.0) | 70.9 (2.7) | 0.38 |
| Types of chronic conditions |  |  |  |
| Arthritis | 20.2 (2.8) | 24.9 (2.5) | 0.20 |
| Asthma | 6.1 (2.5) | 8.9 (1.2) | 0.43 |
| Cancer | 2.8 (1.4) | 5.7 (1.1) | 0.15 |
| Cerebrovascular disease | 4.7 (1.4) | 4.4 (1.0) | 0.85 |
| Congestive heart failure | 2.4 (1.3) | 5.4 (1.2) | 0.15 |
| Chronic renal failure | 2.1 (1.1) | 5.3 (1.1) | 0.10 |
| Chronic obstructive pulmonary disease | 7.5 (1.7) | 13.3 (1.8) | 0.02 |
| Depression | 13.2 (4.5) | 18.1 (2.2) | 0.42 |
| Diabetes | 37.3 (3.7) | 34.0 (2.1) | 0.40 |

| S1 Table (continued). Characteristics of Dual Eligible Individuals with Primary Care Visits, Stratified by Safety-Net Clinic Use | | | |
| --- | --- | --- | --- |
|  | Weighted % (SE) | | p-value |
|  | Safety-Net Clinics  (N = 526) | Non-Safety-Net Clinics  (N = 706) |  |
| Estimated total population | 1,086,000 | 6,538,000 |  |
| Clinical Characteristics (continued) |  |  |  |
| Hyperlipidemia | 25.8 (5.7) | 32.1 (3.2) | 0.33 |
| Hypertension | 69.7 (3.7) | 57.7 (3.0) | 0.01 |
| Ischemic heart disease | 10.0 (3.0) | 8.0 (1.1) | 0.53 |
| Obesity | 14.5 (6.0) | 15.3 (2.2) | 0.89 |
| Osteoporosis | 15.0 (3.2) | 7.6 (1.2) | 0.01 |

S1 TABLE. Characteristics of Dual Eligible Individuals with Primary Care Visits, Stratified by Safety-Net Clinic Use
